# The Role of Extracts of Edible Parts and Production Wastes of Globe Artichoke (*Cynara cardunculus* L. var. *scolymus* (L.)) in Counteracting Oxidative Stress

**DOI:** 10.3390/antiox14010116

**Published:** 2025-01-20

**Authors:** Valentina Laghezza Masci, Irene Mezzani, Enrica Alicandri, William Tomassi, Anna Rita Paolacci, Stefano Covino, Vittorio Vinciguerra, Elisabetta Catalani, Davide Cervia, Mario Ciaffi, Stefania Garzoli, Elisa Ovidi

**Affiliations:** 1 Department for Innovation in Biological, Agro-Food and Forest Systems, Tuscia University, 01100 Viterbo, Italy; laghezzamasci@unitus.it (V.L.M.); irene.mezzani@unitus.it (I.M.); enrica.alicandri@unitus.it (E.A.); william.tomassi@unitus.it (W.T.); arpaolacci@unitus.it (A.R.P.); stefano.covino@unitus.it (S.C.); vincigue@unitus.it (V.V.); ecatalani@unitus.it (E.C.); d.cervia@unitus.it (D.C.); eovidi@unitus.it (E.O.); 2 Department of Drug Chemistry and Technology, Sapienza University, 00185 Rome, Italy

**Keywords:** *Cynara cardunculus* L. var. *scolymus*, byproducts cultivation, polyphenols, VOCs, antioxidant activities, neuroprotective activities

## Abstract

In addition to the immature edible flower heads, the cultivation of globe artichoke (*Cynara cardunculus* L. var. *scolymus* (L.) Fiori) generates substantial quantities of by-products, including leaves, stems, and roots, which constitute potential sources of bioactive compounds and prebiotic dietary fiber. Preserving agricultural biodiversity and promoting socioeconomic development are essential for enhancing domestic production and fostering innovation. In the search for new biomolecules with antioxidant properties, this research focused on a globe artichoke landrace at risk of genetic erosion, still cultivated in the northern part of the Lazio region, known as the “Carciofo Ortano”. To investigate the antioxidant properties of various globe artichoke tissues from the “Carciofo Ortano” landrace, methanolic extracts were prepared from the immature main and secondary flower heads, stems, and leaves of representative genotypes of this landrace. Additionally, extracts were obtained from the same tissues of four landraces/clones included in the varietal platform of the PGI “Carciofo Romanesco del Lazio”, which served as reference genotypes: Campagnano, Castellammare, C3, and Grato 1. The antioxidant properties of these extracts were assessed using FRAP, ABTS, DPPH assays, and total phenolic content (TPC). The stem and secondary flower head extracts of two representative “Carciofo Ortano” genotypes and the Grato 1 clone, which have higher phenolic content, demonstrated the highest antioxidant activity. These extracts were therefore studied for their chemical profile using HPLC-DAD and SPME-GC/MS analysis. Additionally, the same extracts were investigated in vitro for their antioxidant capacity in differentiated SH-SY5Y cells, assessing their effects on ROS levels and the restoration of GSH levels. Furthermore, the in vivo beneficial effects of counteracting oxidative stress were evaluated in high sucrose-fed *Drosophila melanogaster*, as oxidative stress is a typical hallmark of hyperglycemic status. Overall, the results indicated that the edible immature inflorescences of the “Carciofo Ortano” landrace, along with the byproducts of its cultivation, are sources of raw materials containing biomolecules whose properties can be exploited for further applications in the pharmaceutical and medical sectors.

## 1. Introduction

Plants play a pivotal role as sources of bioactive compounds with significant antioxidant properties, and interest in the utilization of natural products has markedly increased in recent years [1]. The protective effects of plant-derived secondary metabolites are attributed to both their direct scavenging activity against reactive oxygen species (ROS) and their ability to induce intracellular antioxidant mechanisms [2]. Indeed, numerous epidemiological studies have shown that diets rich in antioxidants, particularly those derived from fruits, vegetables, and other botanicals, are associated with a decrease in premature mortality from cancer and other chronic diseases [3]. ROS are physiologically produced during metabolic processes, particularly in the electron transport chain. At low concentrations, these molecules are essential for several biochemical functions. The body’s endogenous antioxidant system balances the production of ROS and other free radicals, but excessive accumulation of these unstable molecules, surpassing the cellular defense capacity, leads to oxidative stress, which can result in cell death [4]. Oxidative stress is implicated in cellular toxicity and damage to fundamental biomolecules such as nucleic acids, proteins, and lipids, and it is recognized as a contributing factor in the development of numerous diseases, including diabetes, neurodegenerative disorders, and liver and cardiovascular diseases. As a result, dietary antioxidants have been proposed as potential therapeutic agents to mitigate diseases associated with oxidative stress [5].

The globe artichoke (*Cynara cardunculus* L. var. *scolymus* L.) is a perennial plant native to the Mediterranean region, traditionally cultivated for its large, immature inflorescences (capitula or heads), which are sold fresh or processed industrially into frozen, cooked, canned, or oil-preserved products [6,7]. As a vegetable, the globe artichoke is an important component of the Mediterranean diet and is classified as a “functional food” due to its high content of nutraceutical ingredients, including various bioactive compounds with well-documented antioxidant properties, as well as inulin, fiber, and minerals [8,9,10]. Additionally, artichoke leaves have a long history of use in herbal medicine, particularly as a remedy for liver and gallbladder disorders [11]. Currently, dry leaf extracts are commercially available as liver-protective, hypocholesterolemic, choleretic, and lipid-lowering agents [11,12,13]. Clinical and preclinical studies have confirmed that species within the Cynara genus are among the most effective medicinal plants for treating liver disorders [14,15,16,17]. Furthermore, artichoke leaf extracts have demonstrated antioxidant, anti-inflammatory, anticarcinogenic, diuretic, antibacterial, and antifungal properties [9,11,12]. The health benefits of the artichoke plant are primarily attributed to its high content of phenolic compounds, including hydroxycinnamic acids (predominantly mono- and di-O-caffeoylquinic acids), flavone glycosides (chiefly luteolin, apigenin, and their derivatives), terpenes (mainly sesquiterpene lactones), and anthocyanins (predominantly cyanidin derivatives) [18,19]. These bioactive compounds are highly valued for their potential in preventing several human diseases by reducing oxidative stress [20].

In 2022, global artichoke production reached 1,584,513 tonnes, with a cultivated area exceeding 113,000 hectares [21]. Four circum-Mediterranean countries, Egypt (459,962 tonnes), Italy (378,100 tonnes), Spain (200,070 tonnes), and Algeria (124,305 tonnes), were the leading global producers, collectively accounting for 73.36% of total world production [21]. This highlights the substantial contribution of artichoke cultivation to the agricultural economies of the Mediterranean region. Artichoke is also cultivated in the Near East (Turkey and Iran), South America (Perú, Argentina, and Chile), and the United States (mainly in California). More recently, its cultivation has expanded in China, with a 2022 production of 81,022 tonnes [21].

The artichoke typically exhibits a low harvest index, with 80–85% of the total plant biomass consisting of crop residues [22]. Industrial processing generates substantial waste, primarily bracts and stems discarded during harvesting, which accounts for only 15–30% of the total biomass [22,23]. The majority, consisting predominantly of leaves and stalks (70–85%), remains unused in the field when artichoke is grown as a perennial crop. Due to their high moisture content, these residues are susceptible to microbial growth and pose risks of environmental contamination.

Food waste disposal’s growing environmental and economic challenges have led to increased focus on recovery policies and circular economy strategies to reduce waste and enhance its reuse and valorization [24,25]. These strategies aim to safeguard food security, human health, and the environment by mitigating pollution. Current trends in the circular economy prioritize the production of bioenergy, biocomposite materials, and bioactive compounds, with an emphasis on developing sustainable, cost-effective methodologies for waste management [26,27].

In this context, artichoke by-products are considered a valuable source of various bioactive compounds, similar to those found in the edible flower heads, including phenolic and terpenoid compounds [12,28,29], inulin [12,30], and dietary fiber [12,19]. Notably, the phenolic content in these residues exceeds that of other sources such as grape pomace and carrot peels [31]. Several epidemiological and pharmacological studies have highlighted the health-promoting properties of extracts derived from non-edible parts of globe artichokes, which exhibit antioxidant, hepatoprotective, anti-inflammatory, hypoglycaemic, cardio-, and neuro-protective activities, as well as significant antibacterial and antifungal effects [12,18,19,20]. As a result, the abundant aerial biomass of this species has gained increasing attention as a raw material to produce nutraceuticals, food additives, pharmaceuticals, and cosmetics [9,12,18,19], offering new opportunities for the valorization of artichoke crop residues. Furthermore, previous studies have shown that the quantity and quality of bioactive compounds, particularly polyphenols, in non-edible plant parts are influenced by preharvest factors such as genotype, growth conditions, and phenological stage [29,32,33].

Italy, the site of globe artichoke domestication, holds the greatest biodiversity of this crop, encompassing numerous distinct landraces well adapted to diverse local environments [6,34]. Each region maintains its unique local varieties, which differ in morphological traits and chemical composition, resulting in varied bioactive properties [35,36,37]. Despite this extensive biodiversity, most of the Italian artichoke production relies on a limited number of clones or varieties [37,38,39]. Furthermore, the recent introduction of non-autochthonous, seed-propagated varieties has contributed to the gradual erosion of local landraces [37,39,40], a trend that parallels similar issues affecting local varieties in other European countries [39,41].

As defined by the United Nations Conference on Environment and Development (UNCED) in 1992, the preservation of biodiversity forms the foundation of sustainable development strategies up to 2050, alongside the adoption of circular economy production models [42]. These guidelines underpin the evolving role of agriculture, which has gained recognition for its multifunctionality. Agriculture now provides not only food but also contributes to food security, territorial balance, landscape conservation, and environmental protection [43]. It is increasingly viewed as a vital resource for local communities, fostering connections with other sectors, such as agri-tourism, nature management, and social farming [44]. In addition, as outlined above, from a circular economy perspective, the valorization and reutilization of agricultural waste and by-products is critical, enabling the development of low-impact processes where waste from one operation becomes a resource for another [45].

This research, aimed at safeguarding agricultural biodiversity and promoting a circular economy perspective, is focused on the chemical characterization and antioxidant activity of various plant parts from a globe artichoke landrace still cultivated in the Lazio region (Central Italy), known as the “Carciofo Ortano” [46]. Specifically, the study sought to identify bioactive compounds with antioxidant properties across different tissues of this landrace, with the goal of developing strategies to enhance its value. The combination of in vitro and in vivo studies yielded valuable insights into the biological activity of various plant parts from the “Carciofo Ortano” artichoke landrace, opening the possibility of identifying useful biomolecules that can be exploited for further applications in the pharmaceutical and medical sectors.

## 2. Materials and Methods

### 2.1. Plant Materials

The ex situ field collection site located in the countryside of the Orte municipality (latitude 42°47′ N, longitude 12°33′ E, altitude 130 m), was used for growing globe artichoke plants. The study examined ten distinct genotypes (Table S1). Six of these were chosen as representative genotypes of two populations, namely Orte 1 and Orte 2, identified within the “Carciofo Ortano” landrace [46]. These genotypes were designated as F3 P8, F4 P10, and F17 P3 for the Orte 1 population, and F7 P2, F7 P5, and F15 P3 for the Orte 2 population. The remaining four genotypes, used as controls in the analyses, consisted of four landraces/clones from the varietal platform of the PGI “Carciofo Romanesco del Lazio”: Campagnano, Castellammare, C3, and Grato 1. In November 2020, all artichoke genotypes were vegetatively propagated using offshoots and subsequently transplanted into the open field. The planting arrangement featured a row spacing of 1.00 m and an inter-row spacing of 2.5 m. The experimental design utilized a randomized complete block layout, incorporating three replications per treatment, with each experimental unit consisting of five plants. Field trials were carried out under conditions of restricted resource inputs, which included two annual irrigations in April and August and each delivering 60 mm of water. A nitrogen rate of 50 kg per hectare was applied through organic fertilization and the cultivation practices adhered to local agronomic methods, excluding the use of pesticides, herbicides, and gibberellic acid. During the harvest period of the 2022 growing season, the primary and secondary flower heads (PFHs and SFHs, respectively), along with leaves and stems, were collected from three plants per genotype, with one plant taken for each replicate. Immediately after collection, the plant material was sliced, stored at −80 °C, and subsequently freeze dried. The lyophilized samples were subsequently stored at 4 °C in sealed plastic bags, kept under vacuum, and protected from light until used for analysis. The four distinct plant organs from thirty plants (comprising ten genotypes × three biological replicates) were pooled, resulting in forty samples.

### 2.2. Methanol Extracts and Sample Preparation for HPLC-DAD Analysis

Powdered freeze-dried artichoke plant samples (2.0 g) were subjected to ultrasound-assisted extraction with 60 mL of 100% methanol (200 W) for three cycles of 30 min each at room temperature. Following filtration through 0.45 µm Whatman filter paper to eliminate all solid residues, 15.0 mL of each extract was concentrated by rotary evaporation under reduced pressure at 30 °C, and 20.0 mL of water was subsequently added to the resulting solid residue.

### 2.3. Antioxidant Activity Assays and Total Phenolic Content Analyses

#### 2.3.1. Ferric Reducing Antioxidant Power (FRAP)

The ferric ion reducing antioxidant power (FRAP) assay measures the capacity of antioxidants to reduce Fe^3+^ to Fe^2+^. The Fe^2+^ concentration was quantified spectrophotometrically by assessing the formation of a colored complex with 2,4,6-Tris (2-pyridyl)-s-triazine (TPTZ), which exhibits high absorption at 595 nm. The microplate FRAP assay was performed following the method outlined by Xiao et al. [47]. To prepare the FRAP working solution, 10 mL of 300 mM acetate buffer (pH 3.6, adjusted with acetic acid), 1 mL of 20 mM ferric chloride hexahydrate 20 mM dissolved in distilled water, and 1 mL of 2,4,6-Tris(2-pyridyl)-s-triazine (TPTZ) 10 mM dissolved in 40 mM HCl were mixed freshly.

In 96-well microplates, 20 μL of the appropriate concentration of samples and Trolox dilutions were mixed with 180 μL of the FRAP solution. The absorbance at 595 nm was measured using a Tecan Sunrise^TM^ UV–Vis spectrophotometer (Tecan Group Ltd., Männedorf, Switzerland) after incubation for 40 min at 37 °C. Trolox solutions, ranging from 2 to 0.002 mM, were used for standard curve. Three different experiments were carried out.

Results were reported as μmol TE/g DW according to the formula of [48]:

FRAP value (μmol TE/g DW) = *c* × *V* × *t*/*m*

where *c* is the Trolox concentration (μmol/mL) of the corresponding standard curve of the diluted sample, *V* is the sample volume (mL), *t* is the dilution factor, and *m* is the weight of the sample dry matter (g).

#### 2.3.2. ABTS

The free radical-scavenging capacity of all samples was evaluated using the 2,2′-azinobis-(3-ethylbenzothiazoline-6-sulfonic acid) diammonium salt (ABTS) radical assay, following the procedure described by Xiao et al. [47]. To generate ABTS^•+^ radical cation, 7 mM of ABTS and 2.45 mM of potassium persulfate were mixed and incubated in the dark at room temperature for at least 16 h. The resulting working solution was then diluted with methanol (MeOH) to achieve an absorbance of 0.70 ± 0.02 at 734 nm. Subsequently, 180 μL of diluted ABTS^•+^ solution was combined with 20 μL of sample extracts and incubated at room temperature for 6 min in the dark.

The absorbance was measured at 734 nm using the Tecan Sunrise^TM^ UV–Vis spectrophotometer (Tecan Group Ltd., Männedorf, Switzerland). Different concentrations of Trolox (ranging from 2 to 0.002 mM) were used to draw the standard curve. The results were obtained in triplicate and expressed as μmol TE/g DW as previously described.

#### 2.3.3. DPPH Radical Scavenging Activity

The antioxidant activity of globe artichoke sample extracts was also assessed using the DPPH (2, 2-diphenyl-1-picrylhydrazyl) radical scavenging microplate method, as outlined by Xiao et al. [47]. In this procedure, 20 μL of the diluted sample, prepared at various concentrations, were added to 180 μL of a 0.15 mM DPPH solution in a 96-well microplate. After incubating the mixture in the dark at room temperature for 40 min, the absorbance was measured at 515 nm using a Tecan Sunrise^TM^ UV–Vis spectrophotometer (Tecan Group Ltd., Männedorf, Switzerland). Trolox was used as a standard at different concentrations to generate the standard curve. As previously described, the results were obtained in triplicate and expressed as μmol TE/g DW.

#### 2.3.4. Phenolic Content (TPC)

Total Phenolic Content (TPC) was assessed using the Folin–Ciocalteu colorimetric method [48] with slight modifications [46]. In brief, methanol extracts ranging from 0.01 to 5 mg of dry weight per mL were mixed with 100 µL Folin–Ciocalteu reagent and vigorously shaken. Following the addition of 80 µL of 7.5% Na_2_CO_3_ and a 2 h incubation at room temperature in darkness, absorbance was measured at 765 nm using an Ultrospec 1000 spectrophotometer (Phamacia Biotech, Uppsala, Sweden). The results were obtained in triplicate and expressed as mg gallic acid equivalents (GAE)/g DW.

### 2.4. Chemical Analyses

#### 2.4.1. HPLC-DAD Analysis

Polyphenols of artichoke plant extracts were analyzed using the same procedure, instrumentation and conditions described by Laghezza Masci et al. [49].

For the purification and fractionation of the samples, 4.0 mL of the aqueous supernatant, obtained from the extraction process and reconstituted with water to the resulting solid residue, was loaded onto a C18 reversed-phase cartridge, which had been pre-activated with methanol and rinsed with water. The cartridge was then eluted sequentially, first with 10% aqueous methanol to isolate fraction I (phenolic acids), followed by methanol to obtain fraction II (neutral compounds). The eluates were evaporated to dryness under vacuum, and the resulting residues were reconstituted in 1.0 mL of 50% aqueous methanol for fraction II and 4.0 mL of 50% aqueous methanol for fraction I.

A Shimadzu “Prominence” system equipped with an SPD-M20A diode array detector was used, and separation was performed on a C18 Hyperchrom Bischoff column (250 × 4.0 mm i.d., 5 μm particle size) at 35 °C. The mobile phase consisted of 2% acetic acid in water (eluent A) and 0.5% acetic acid in water and acetonitrile (50:50, *v*/*v*; eluent B). A gradient was applied over 90 min as follows: starting at 10% B, increasing to 18% B over 20 min, then to 24% B over the next 10 min, and further to 30% B over 15 min. The concentration of B was maintained at 30% for 20 min, followed by an increase to 55% B over 5 min. This was then ramped up to 100% B over 5 min, with the final condition held at 100% B for 8 min. Samples were injected in a volume of 20.0 μL, and chromatograms were recorded at 320 nm for hydroxycinnamic acids, 330 nm for apigenin derivatives, 350 nm for luteolin derivatives, and 232 nm for cynaropicrin, with a flow rate of 0.8 mL/min.

Compound identification was achieved by comparing retention times and UV spectra with those of known commercial standards. For quantitative analysis, standard calibration curves were employed, with chlorogenic acid serving as the reference for mono-O-caffeoylquinic acids and cynarin for di-O-caffeoylquinic acids. Derivatives of apigenin and luteolin were quantified as apigenin 7-O-glucoside and luteolin 7-O-glucoside, respectively. The quantification of cynaropicrin was performed using an external standard based on a commercial cynaropicrin calibration curve.

#### 2.4.2. SPME Sampling

The volatile fraction of the matrices was collected using the solid phase microextraction (SPME) technique. The operational conditions followed those described by Laghezza Masci et al. [49]. Briefly, approximately 2 g of each sample were placed in a 7 mL glass vial with a PTFE-coated silicone septum. For the adsorption of components, a SPME device from Supelco (Bellefonte, PA, USA) with a 1 cm fiber coated with 50/30 μm DVB/CAR/PDMS (divinylbenzene/carboxen/polydimethylsiloxane) was used. The fiber was reconditioned between samples to remove residual traces from previous extractions. After reaching the equilibrium phase, the fiber was exposed to the headspace of the samples for 30 min at 40 °C to capture and pre-concentrate the volatiles. Lastly, the analytes were thermally desorbed in the GC injector held at 250 °C for 2 min in spitless mode.

#### 2.4.3. GC-MS Analysis

A Clarus 500 model gas chromatograph (Perkin Elmer, Waltham, MA, USA), equipped with flame detector ionization (FID) and coupled with a mass spectrometer, was used for the analyses. A Varian Factor Four VF-1 capillary column was installed in the GC oven. The oven temperature was initially held at 50 °C and then increased at a rate of 6 °C/min to 220 °C, where it was maintained for 10 min. Helium was used as the carrier gas at a constant flow rate of 1 mL/min. The mass spectrometry (MS) conditions were as follows: ion source temperature was set at 180 °C, electron energy was 70 eV, quadrupole temperature was maintained at 200 °C, the GC-MS interface was kept at 220 °C; and the scan range was from 35 to 450 mass units. Compound identification was achieved by comparing their mass spectra with those of pure components from the Wiley 2.2 and Nist 11 spectral libraries, as well as by comparing Linear Retention Indices (LRIs) calculated using a series of alkane standards (C_8_–C_25_ *n*-alkanes) with literature values. The relative amounts of the identified components were expressed as percentages extrapolated from FID peak-area normalization (mean of three replicates), without the use of an internal standard or any factor corrections.

### 2.5. Antioxidant Activity Assay on Cell Culture

The artichoke plant extracts from different genotypes, which showed higher antioxidant activity as measured by FRAP, ABTS, and DPPH assays, along with higher total phenolic content, were further evaluated for their ability to protect against oxidative stress by measuring intracellular reactive oxygen species (ROS) and reduced glutathione (GSH) levels in an in vitro system represented by a differentiated SH-SY5Y cell line. Stock solutions of the dried extracts were prepared by dissolving them in dimethyl sulfoxide (DMSO).

#### 2.5.1. SH-SY5Y Cell Viability

Human neuroblastoma cells (SH-SY5Y, ATCC^®^ CRL-2266) were cultured in DMEM F-12 supplemented with 10% (*v*/*v*) fetal bovine serum (FBS), 1% of L-glutamine, 1% of penicillin/streptomycin, and maintained at 37 °C in a humidified incubator with 5% CO_2_. Once the cells reached their logarithmic growth phase, differentiation into a neuronal phenotype was induced with 10 µM retinoic acid (RA) for 7 days. Differentiated SH-SY5Y cells were plated in a 96-well culture plate and incubated at 37 °C with 5% CO_2_ for 24 h to facilitate attachment of the cells to the bottom of the wells. Then, the cells were exposed to ten two-fold serial dilutions (ranging from 0.5 to 250 µg/mL) of stem and SFH methanolic extracts of three different globe artichoke genotypes: Orte 1 F4 P10, Orte 2 F7 P2 and Grato 1. The highest DMSO treatment (0.1% DMSO solution) was used as a solvent control. The media containing the extracts was removed, and 0.5 mg/mL of MTT in culture medium was added. Following 3 h of incubation, the MTT solution was discarded, and DMSO was introduced to solubilize the purple-colored formazan product. Absorbance was measured at 595 nm using a microplate reader (Tecan Sunrise^TM^ UV–Vis spectrophotometer). The half-maximal effective concentration (EC_50_) for each biological replicate was determined using a log-response curve, with the EC_50_ values calculated from three independent biological experiments [50].

To evaluate the ability of stem and SFH extracts from the three different genotypes to protect against oxidative stress, five no-cytotoxic concentrations diluted one to two were used (0.39 to 6.25 µg/mL for stem extracts and 12.5 to 200 µg/mL for SFH extracts). After 24 h, oxidative stress was induced by exposing the cells to 700 μM H_2_O_2_ for 1 h. Cell viability was then assessed by measuring MTT reduction, as previously described.

#### 2.5.2. Intracellular ROS Measurement

Intracellular ROS formation was assessed using the fluorescent DCFH-DA probe. In brief, differentiated SH-SY5Y cells were treated for 24 h with 6.25 μg/mL and 200 μg/mL of stem and SFH extracts, respectively. Subsequently, the cells were incubated with 10 μM of DCFH-DA in DMEM 1% FBS w/o phenol red for 30 min. Following the removal of DCFH-DA, the cells were incubated with 400 μM H_2_O_2_ in DMEM 1% FBS w/o phenol red for 15 min. The H_2_O_2_ was then removed and replaced with PBS. Fluorescence was then measured at 485 nm (excitation) and 535 nm (emission) with a Viktor X3 multilabel plate reader (PerkinElmer, Waltham, MA, USA).

#### 2.5.3. Determination of Reduced Glutathione (GSH) Levels

Reduced glutathione (GSH) levels were determined using the monochlorobimane (MCB) fluorometric assay, as described by Angeloni et al. [51]. Prior to oxidative stress induction with 700 μM H_2_O_2_ for 1 h, the differentiated SH-SY5Y cells were treated as previously reported for the intracellular ROS measurement. Following treatment, the cells were incubated with 50 μM MCB 1% FBS w/o phenol red for 30 min at 37 °C. After incubation, fluorescence was measured at 355 nm (excitation) and 460 nm (emission) using a VICTOR2 D fluorometer (PerkinElmer, Waltham, MA, USA) multilabel plate reader.

### 2.6. Mitochondrial Viability Assessment in D. melanogaster Head Homogenate

As previously described for similar experiments [52], adult (5–6 days old) female and male *D. melanogaster* (Oregon-R strain from Bloomington Drosophila Stock Center, Indiana University, Bloomington, IN, USA) were reared for 10 days in vials containing either a standard diet (STD) or high-sucrose diet (HSD, 35% *w*/*v*). The HSD was prepared by independently varying the sucrose concentration while keeping all other components constant. Where indicated, flies were raised on diets supplemented with SFH or stem extracts at a concentration ranging from 0.03 to 0.1 mg/mL, ensuring that the DMSO concentration remained below 0.1% to avoid poisoning (toxic) effects. Compounds were mixed into the cooled food in the vials to achieve the final working concentration protected from light.

Mitochondrial viability in *D. melanogaster* head homogenate was assessed, as previously described [53,54], with minor modifications. In brief, 100 heads were manually homogenized in cold Phosphate Buffer (PB). The supernatants were collected after each consecutive centrifugation at 4 °C for 5 min at 1000 rpm. Mitochondrial activity was subsequently evaluated using the MTT reduction assay, with a final concentration of 0.5 mg/mL, by measuring absorbance at 595 nm.

### 2.7. Statistical Analyses

Statistical analyses were conducted using JMP PRO 15 (Trial Version, SAS Institute Inc., Cary, NC, USA) and GraphPad Prism 5.0 (GraphPad Software, San Diego, CA, USA). One-way analysis of variance (ANOVA), followed by Tukey’s post hoc test for multiple comparisons, was employed to determine significant differences among groups. Additionally, Principal Component Analysis (PCoA), and Pearson’s correlation coefficient were applied to assess data correlations. Statistical significance was defined as a *p*-value ≤ 0.05. Results are expressed as means ± standard deviation (SD) or standard error of the mean (SEM), as indicated by the corresponding sample size (n).

## 3. Results and Discussion

### 3.1. Antioxidant Activities and Total Phenolic Content in Different Tissues of Artichoke Genotypes

The antioxidant activities of methanolic extracts from four artichoke tissues (leaf, stem, primary, and secondary flower heads) were evaluated using four different in vitro assays: FRAP, ABTS, DPPH, and Folin–Ciocalteu (TPC). Although the latter assay is predominantly employed to quantify total phenolic content, it also responds to all oxidizable groups interacting with the reagent. Therefore, it serves as an indicator of the overall reduction capacity, which directly correlates with phenolic content and antioxidant capacity [55].

Six representative genotypes of the “Carciofo Ortano” landrace were included in the analyses: three from the Orte 1 population (F4 P10, F3 P8, and F17 P3) and three from the Orte 2 population (F7 P2, F7 P5, and F15 P3). Additionally, four landraces/clones from the varietal platform of the PGI “Carciofo Romanesco del Lazio” (Campagnano, C3, Grato 1, and Castellammare) were used as reference genotypes. The aim was to select the genotypes and tissues within each of the three groups (Orte 1 and Orte 2 populations, and reference genotypes) that exhibited higher antioxidant activity for further characterization.

ANOVA was conducted individually for each genotype group across the four assays, unveiling notable variations in antioxidant activities among the tissues and genotypes within each specific group (Table 1, Table 2, Table 3 and Table 4). However, a general trend was observed across the four tissues, regardless of the genotype group. Specifically, the highest antioxidant activities and phenol content, as measured by the four assays, were generally found in the stem and secondary flower head (SFH), closely followed by the primary flower head (PFH), while significantly lower values were observed in the leaves (Table 1, Table 2, Table 3 and Table 4).

For the FRAP assay, in the Orte 1 population, Orte 1 F4 P10 exhibited the highest values in the SFH (407.52 µmol TE/g DW), which was not significantly different from what was observed in Orte 1 F3 P8 in the stem (400.26 µmol TE/g DW). In contrast, Orte 1 F17 P3 showed significantly lower FRAP values in both the stem (211.29 µmol TE/g DW) and SFH (254.76 µmol TE/g DW), as well as in all other tissues analyzed, compared to the other two genotypes (Table 1).

In the Orte 2 population, the highest antioxidant activities, as assessed using the FRAP method, were observed in the stems of the Orte 2 F15 P3 (552.57 µmol TE/g DW) and Orte 2 F7 P2 (519.67 µmol TE/g DW) genotypes. Notably, the latter genotype also demonstrated significantly elevated antioxidant activity in the SFH (434.14 µmol TE/g DW) (Table 1). In contrast, the Orte 2 F7 P5 genotype exhibited significantly lower values across all tissues analyzed when compared with the other two genotypes.

Among the control genotypes, Grato 1 exhibited the highest antioxidant activity in the SFH (471.59 µmol TE/g DW). Moreover, significantly elevated FRAP values (>400 µmol/g DW) were observed in the PFHs of Campagnano, Grato 1, and C3, as well as in the SFH and stem tissues of C3 (Table 1).

For the Orte 1 population, the highest ABTS values were observed in the stem (380.60 µmol TE/g DW) and SFH (372.16 µmol TE/g DW) tissues of Orte 1 F4 P10. These values, however, were not significantly different from those recorded in the SFH of Orte 1 F17 P3 (377.76 µmol TE/g DW) and Orte 1 F3 P8 (357.76 µmol TE/g DW). Nonetheless, the latter genotypes exhibited significantly lower antioxidant activity in the stem compared to Orte 1 F4 P10 (Table 2). In the Orte 2 population, the highest level of antioxidant activity, as measured by the ABTS assay, was recorded in the stem of the Orte 2 F7 P2 genotype (451.64 µmol TE/g DW), which was significantly greater than the values observed in all other analyzed sample tissues (Table 2). Notably, in addition to the stem, Orte 2 F7 P2 exhibited the highest values across all the three remaining tissue types when compared to the other two genotypes in the Orte 2 population (Table 2).

Among the reference genotypes, Grato 1 exhibited some of the highest values for antioxidant activity, as measured by the ABTS assay, in both the stem (388.06 µmol TE/g DW) and SFH (381.61 µmol TE/g DW) tissues. These values were not significantly different from those detected in the PFH of Campagnano (391.27 µmol TE/g DW) or the SFH of Castellammare (360.03 µmol TE/g DW) (Table 2). Notably, appreciable antioxidant activity was also observed in the PFH of Grato 1 (335.08 µmol TE/g DW) and in the stem of Campagnano (324.98 µmol TE/g DW) (Table 2).

In the DPPH assay, within the Orte 1 population, the Orte 1 F4 P10 genotype exhibited the highest antioxidant activity in the SFH (1232.56 µmol TE/g DW), which was significantly higher than that of the other genotypes across all the examined tissues (Table 3). Additionally, this genotype also showed notable antioxidant activity in the PFH (1075.91 µmol TE/g DW), highlighting its strong antioxidant potential in the flower heads. Appreciable levels of antioxidant activity, as measured by the DPPH assay, were also detected in the PFH (1164.42 µmol TE/g DW), stem (1009.10 µmol TE/g DW), and SFH (1017.68 µmol TE/g DW) tissues of the Orte 1 F3 P8 genotype (Table 3).

In the Orte 2 population, Orte 2 F15 P3 demonstrated an exceptionally high DPPH value in the stem (1685.71 µmol TE/g DW), which was significantly higher than that of the other genotypes across all the analyzed tissues. Notable antioxidant activity, as measured by the DPPH assay, was also recorded in the SFH (1591.16 µmol TE/g DW) and stem (1297.61 µmol/g DW) tissues of the Orte 2 F7 P2 genotype (Table 3).

Among the reference genotypes, the highest antioxidant activity was detected in the SFH (1706.41 µmol TE/g DW) and stem (1508.29 µmol TE/g DW) tissues of Grato 1. Castellammare also showed notable DPPH activity, registering some of the highest values in both PFH (1373.56 µmol TE/g DW) and SFH (928.15 µmol TE/g DW) tissues (Table 3).

When considering the total polyphenol content (TPC), it is important first to emphasize the notably high concentration of these compounds in the tissues of the three genotypes from the Orte 2 population, especially in the stems, as compared to the levels detected in the other two genotype groups (Orte 1 population and reference genotypes) (Table 4).

In the Orte 1 population, the highest TPC was detected in the SFH (154.27 ± 1.59 mg GAE/g DW) and stem (92.84 ± 1.76 mg GAE/g DW) tissues of the Orte 1 F4 P10 genotype (Table 4). Notable total phenolic content was also recorded in the PHF (88.81 ± 1.19 mg GAE/g DW) and SFH (79.01 ± 1.25 mg GAE/g DW) of Orte 1 F17 P3, while Orte 1 F3 P8 exhibited significantly lower TPC across all tissues analyzed, when compared with the other two genotypes within the Orte 1 population (Table 4).

In the Orte 2 population, the Orte 2 F7 P2 genotype recorded the highest TPC in the stem (245.56 mg GAE/g DW), which was not significantly different from the value detected in the same tissue for the Orte 2 F15 P3 genotype (242.17 mg GAE/g DW) (Table 4). The SFH consistently represented the second tissue with the highest TPC levels within this population, with the Orte 2 F7 P2 and Orte 2 F15 P3 genotypes again displaying the highest values in this tissue (183.44 and 173.56 mg GAE/g DW, respectively) (Table 4).

Among the reference genotypes, the highest TPC values were recorded in the PFHs of Campagnano and C3, measuring 126.30 and 120.36 mg GAE/g DW, respectively. However, when considering the other three tissues, Grato 1 exhibited significantly higher TPC values compared to the other three reference genotypes, with notable levels detected in the SFH (97.59 mg GAE/g DW) and stem (92.09 mg GAE/g DW) tissues (Table 4).

The investigation into the antioxidant abilities of methanolic extracts from the leaf, stem, PFH and SFH of the “Carciofo Ortano” genotypes revealed significant antioxidant potential in the various tissues of this landrace. It also confirmed the high antioxidant capacity of the artichoke plant, which exhibited higher ABTS, FRAP, and TPC values than other vegetables such as cucumber, asparagus, cabbage, red beet, radish, and turnip [19]. This high antioxidant capacity is primarily found in the flower heads and leaves [56], although other artichoke by-products may also possess higher antioxidant potential than many other foods [19]. In this study, the highest values obtained in all the antioxidant assays used were detected in methanolic extracts from the stem (for FRAP, ABTS and TPC assays) and from SFH (for the DPPH assay).

Several studies have highlighted significant variation in the antioxidant properties of artichoke, which can be attributed to differences in extraction methods and the plant parts used, including by-products and waste from its cultivation [57]. Methanolic extracts from the edible primary heads of two globe artichoke varieties, namely “*Spinoso Sardo*” and “*Romanesco Siciliano*”, exhibited antioxidant activities comparable to those observed in the reference genotypes included in the varietal platform of the PGI “Carciofo Romanesco del Lazio” in the present study. The antioxidant values for the two varieties were 274.9–467.7, 145.5–287.1, and 530.8–831.3 μmol TE/g DW for the DPPH, ABTS, and FRAP assays, respectively [58], which, however, were generally lower than those obtained for the “Carciofo Ortano” genotypes. On the other hand, DPPH values from the primary and secondary heads in all the genotypes analyzed in this study were very high compared to the value (128 μmol TE/g DW) reported by Jimenez-Escrig et al. [59] for the edible head of Spanish commercial artichoke. Galieni et al. [36] conducted a comprehensive study demonstrating the radical scavenging activity of the external bracts and receptacle of primary and secondary flower heads from ten different Italian artichoke genotypes using ABTS and DPPH assays. The ABTS values ranged from 21.2 to 146.8 μmol TE/g DW and the DPPH values ranged from 12.8 to 204.3 μmol TE/g DW, both of which were significantly lower than those that were detected, across the various tissues, in the genotypes analyzed in the present study. The research found that the highest antioxidant activity was detected in the receptacle compared to the external bracts, with average values of 85.7 and 103.8 μmol TE g^−1^ DW for ABTS and DPPH, respectively, in the receptacle, and 41.9 and 38.3 μmol TE g^−1^ DW for ABTS and DPPH, respectively, in the external bracts. In contrast to the above, Shallan et al. [60] revealed a pronounced capacity of ethanol extracts from external bracts to scavenge DPPH radicals compared to the receptacle, with IC_50_ values of 6.42 and 28.2 μg/mL respectively. However, the same study also highlighted the exceptional antioxidant capacity of the receptacle, which exhibited a FRAP value of 493.9 μmol TE/mL, emphasizing its significant ability to counteract oxidative stress. Additionally, ethanol extracts from the receptacle demonstrated remarkable efficacy in reducing ferric ions, with a value of 527.79 μmol Fe^2+^ per milligram of dry extract. An interesting study done on artichoke waste has highlighted differences in values obtained with different tests such as FRAP, DPPH and ABTS, in their results it was observed how the same extract shows different values but with the same trend indicating that their antioxidant activity is probably regulated by the same constituents [61].

The correlation analysis was conducted using the results from the four antioxidant assays performed on forty methanolic extracts (10 genotypes × 4 tissues) with the Pearson test, to identify which assays produced comparable results and which differed (Table 5). This test calculates the linear correlation coefficient (r), a dimensionless value ranging from −1 to 1, inclusive, which quantifies the strength and direction of a linear relationship between two datasets. A coefficient closer to either extreme indicates a stronger positive or negative correlation, respectively, while a value of 0 signifies no linear correlation. The highest significant correlation (r = 0.725) was found between the ABTS assay and total polyphenol content (TPC) measured by the Folin–Ciocalteu assay (FC). The FRAP assay also demonstrated a strong correlation with TPC (r = 0.679) and the ABTS assay (r = 0.659). In contrast, the DPPH assay exhibited lower, although significant, correlations with ABTS (r = 0.449), TPC (r = 0.416), and FRAP (r = 0.401).

Wootton-Beard et al. [62] reported comparable findings in their investigation of the antioxidant capacity and total polyphenol content of vegetable juices. They observed strong positive correlations between the FRAP, FC, and ABTS assays, with coefficients of determination (R^2^) > 0.9. In contrast, weaker correlations were found between DPPH and ABTS (R^2^ = 0.33), DPPH and FC (R^2^ = 0.44), and DPPH and FRAP (R^2^ = 0.45). Similarly, Zhang et al. [63] evaluated the antioxidant capacities of flavonoids using the four assays and identified the strongest correlation between FRAP and FC (R^2^ = 0.96) and the weakest between DPPH and ABTS (R^2^ = 0.47). Additionally, they calculated bond dissociation enthalpies to evaluate hydrogen-atom-donating abilities and ionization potentials to assess the scavenging activity of the tested compounds. Their findings demonstrated that the DPPH assay aligns more closely with the hydrogen atom transfer (HAT) mechanism compared to the other assays. This observation is further supported by the fact that the FRAP assay, as a non-radical, single-electron transfer (SET)-based method, exhibits limited association with the HAT mechanism. Consequently, the FRAP assay is recommended to be used in combination with other methods to effectively distinguish the dominant antioxidant mechanisms of various compounds. Collectively, these results support our findings, indicating that FRAP, FC, and ABTS exhibit strong positive inter-correlations (r > 0.65), while the DPPH assay shows only weak correlations (r < 0.45) with the other methods.

Nevertheless, these observations are not universally applicable and depend on the nature and composition of the sample under scrutiny. Indeed, other studies, using different food and plant matrices, including various artichoke plant parts, have revealed strong correlations (r > 0.9) across all four assays [58,64,65,66,67]. This highlights the importance of conducting a comprehensive evaluation of each assay to determine its suitability for the specific sample type under investigation. However, employing a range of assays that are less closely related to one another may provide a deeper insight into the key complex antioxidant mechanisms, rather than relying solely on a single test.

### 3.2. Principal Component Analysis

To analyze comprehensively the changes in antioxidant activities influenced by genotype and different plant tissues, a principal component analysis (PCA) was performed on the entire dataset (data obtained from four antioxidant assays in 40 methanolic extracts). Specifically, the PCA was conducted to identify the representative genotypes and tissues within each of the three genotype groups (Orte 1 and Orte 2 populations, and reference genotypes) that exhibited the most significant antioxidant activities (FRAP, ABTS, DPPH) and total phenolic content (TPC).

The first two principal components (PC1 and PC2) cumulatively accounted for a total explained variance of 82.05% (Table S2). Specifically, PC1 explained 65.62% of the total variance, and included contributions from the ABTS and FRAP assays, along with TPC (Table S2). PC2, on the other hand, explained 16.43% of the total variance and was correlated with the DPPH assay (Table S2).

**Figure 1:**
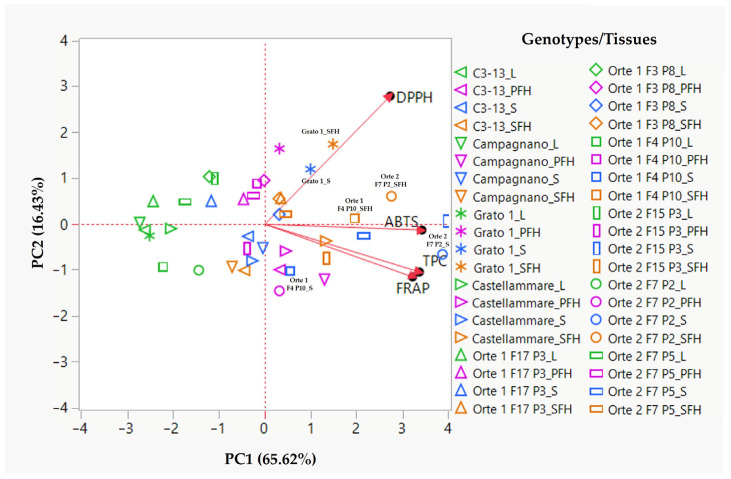
PCA biplot on antioxidant activities (FRAP, ABTS, DPPH) and total phenolic content (TPC) across the different genotypes and tissues. In the biplot, leaf tissues are represented in green, primary flower heads (PFH) in purple, secondary flower heads (SFH) in orange, and stem tissues in blue.

The PCA scores effectively separated the different samples into distinct groups, facilitating the interpretation of results based on all the examined antioxidant assays. Based on PC1, which explained the larger part of the total variance, it is possible to distinguish two distinct groups of samples with negative and positive PCA scores on the left and right sides of the PCA biplot (Figure 1). The first group included samples with lower antioxidant activities, as measured by ABTS, FRAP, and TPC, and was represented by all leaf samples and predominantly by the PFH samples. The second group comprised samples with higher ABTS, FRAP, and TPC values, represented mainly by SFH and stem samples. On the other hand, PC2 distinguished between these two main groups by discriminating samples with higher and lower DPPH values, located in the upper and lower parts of the graph, respectively (Figure 1).

These results clearly indicated that, in most genotypes, the highest antioxidant activities and total phenolic content were found in the stem and SFH tissues among the four plant tissues tested. This is consistent with information available in the literature regarding the antioxidant properties of different artichoke plant parts. For example, studies by Pandino and colleagues [68,69] analyzing two artichoke germplasm collections, comprising cultivated and wild *Cynara cardunculus* genotypes, showed that the stem exhibited higher antioxidant capacity, measured by the FRAP and DPPH assays, compared to other artichoke plant parts, including leaves, receptacles, and inner and outer bracts. Furthermore, Lutz et al. [70] reported that the secondary flower heads of the cultivar Green Globe demonstrated higher scavenging capacity, as measured by the DPPH assay, than the primary flower heads.

The PCA biplot also facilitated the selection of genotypes and tissues exhibiting the highest antioxidant activities by considering their close association with the direction and strength of the eigenvectors of the four antioxidant assays. Within the three genotype groups, Orte 1 F4 P10, Orte 2 F7 P2, and Grato 1 emerged as the most promising for their antioxidant activities, especially in the stem and secondary flower head (SFH) tissues, confirming the results of the ANOVA (Table 1, Table 2, Table 3 and Table 4). Specifically, in both the stem and SFH tissues of Orte 2 F7 P2, and in the SFH of Orte 1 F4 P10, a close alignment with the ABTS and TPC eigenvectors was detected (Figure 1), indicating high phenolic content and ABTS radical scavenging ability in these genotypes and tissues. Orte 1 F4 P10 also exhibited significant antioxidant capacity in the stem, which was well-represented along the FRAP eigenvector, confirming its high reducing power. Finally, the SFH and stem tissues of Grato 1 were strongly correlated with the DPPH eigenvector (Figure 1), indicating their highest radical scavenging activity, consistent with their high DPPH values.

The high phenolic content and strong antioxidant activities observed in the SFH and stem tissues of the Orte 1 F4 P10, Orte 2 F7 P2, and Grato 1 genotypes, as indicated by their strong associations with the first two principal components and corresponding eigenvectors, validate their selection as representative genotypes from the three groups for in-depth chemical and biological profiling.

### 3.3. Chemical Investigation

#### 3.3.1. Characterization and Quantification of Phenolic Compounds by HPLC-DAD

In the analyzed tissues from the three genotypes, nine hydroxycinnamate and flavone compounds, along with one sesquiterpene lactone (cynaropicrin), were identified based on chromatographic retention times and UV spectrum profiles (Table 6). In all analyzed samples, caffeoylquinic acids were the predominant phenolic compounds, with the greatest contribution attributed to 5-O-caffeoylquinic acid (chlorogenic acid) and 3,5- and 1,5-di-O-caffeoylquinic acids, which were consistently detected in the highest concentrations across genotypes and plant tissues (Table 6). In contrast, extremely small quantities of flavonoids, represented by apigenin and its glycosylated form, apigenin 7-O-glucoside, were detected in only some of the analyzed samples (Table 6).

Although the qualitative phenolic profile was similar across all samples, the total phenolic content, calculated as the sum of individual phenolic compounds, varied significantly between genotypes and plant parts (Table 6). Among the genotypes studied, Orte 2 F7 P2 exhibited the highest total phenolic content in both the stem and SFH (87,849.52 and 55,951.70 mg/kg DW, respectively), followed by Orte 1 F4 P10, which contained a similar total polyphenol content in both the stem (28,571.68 mg/kg DW) and SFH (26,117.05 mg/kg DW). In contrast, Grato 1 displayed the lowest values in both plant tissues (24,125.88 mg/kg DW in the stem and 14,637.16 mg/kg DW in the SFH). These differences can be attributed primarily to the significantly higher amounts of di-O-caffeoylquinic acids, particularly 1,5- and 3,5-di-O-caffeoylquinic acids, detected in the two plant tissues of Orte 2 F7 P2 compared to the other genotypes. Notably, the levels of these two di-O-caffeoylquinic acids were approximately four and three to seven times higher in the stem and SFH of Orte 2 F7 P2, respectively, compared to the same tissues in the other two genotypes. Minimal quantities of 1,3-di-O-caffeoylquinic acid (cynarin) were detected in all samples, with an apparent absence in the SFH of Grato 1 (Table 6). In contrast to the accumulation of di-O-caffeoylquinic acids, the levels of mono-O-caffeoylquinic acids, particularly chlorogenic acid and caffeic acid, exhibited an opposite trend across the three genotypes. Generally, higher amounts were detected in both the stem and SFH of Orte 1 F4 P10, followed by the reference genotype Grato 1, while Orte 2 F7 P2 showed the lowest quantities in both tissues (Table 6).

Among flavonoids, apigenin 7-O-glucoside was detected in minimal amounts in the SFH of Orte 1 F4 P10 (166.17 mg/kg DW) and Orte 2 F7 P2 (90.38 mg/kg DW), but not in the stems of either genotype. This apigenin derivative was completely absent in Grato 1. Moreover, extremely small quantities of apigenin were recorded only in the SFH of Orte 2 F7 P2 (44.48 mg/kg DW) (Table 6).

Finally, regarding cynaropicrin, its concentrations varied significantly among genotypes and tissues (Table 6). Orte 2 F7 P2 showed a significantly higher level of this sesquiterpene lactone in the stem (2254.58 mg/kg DW), followed by the secondary flower head (976.85 mg/kg DW), while lower amounts were detected in both tissues of Orte 1 F4 P10 (740.99 mg/kg DW in SFH and 283.97 mg/kg DW in the stem, respectively). In contrast, Grato 1 showed no detectable cynaropicrin in either tissue (Table 6).

The quantitative and qualitative profiles of polyphenols in various artichoke plant parts are well documented in the literature, revealing that these profiles are influenced by multiple factors. These include the methodologies employed for extraction and analysis, the genotype of the plant, environmental conditions, agronomic practices, and the specific physiological stage of the plant material under investigation [28,29,67,71,72,73,74].

In this study, the total phenolic content, calculated as the sum of individual phenolic compounds, differed significantly among the three analyzed genotypes across the two tissues examined. The content ranged from 24.12 to 87.85 g/kg DW in the stem and from 14.64 to 55.95 g/kg DW in the secondary flower head, with caffeoylquinic acids being the predominant phenolic compounds in both plant parts (Table 6). Pandino and coworkers [29], through the analysis of several clones selected from the two Sicilian landraces “Spinoso di Palermo” and “Violetto di Sicilia”, reported total phenolic content values for stem methanolic extracts, measured by HPLC-DAD, ranging from 2.05 to 12.70 g/kg DW. These values were significantly lower than those that were obtained in this study for the same tissue across all three analyzed genotypes. Furthermore, Lombardo et al. [75] reported very low phenolic content values in methanolic extracts from the edible parts of mature inflorescences of six varieties, ranging from 3.01 to 6.6 g/kg DW, if compared to those observed in this study for the secondary flower heads. Additionally, although the edible parts of mature inflorescences in most varieties contained a notable amount of caffeoylquinic acids, the predominant phenolic compounds were flavonoids, primarily apigenin and its derivatives. These qualitative and quantitative differences may be attributed to the diversity of genotypes analyzed, the varying environmental conditions in which they were grown, and the different extraction conditions used for recovering the phenolic compounds. In this study, an ultrasound-assisted method was employed, in contrast to conventional techniques that involve shaking plant material with alcoholic solvents. Ultrasound treatment typically enhances the extraction of bioactive compounds by leveraging the cavitational effect, which facilitates the release of extractable compounds and promotes mass transport through diffusion and disrupts plant cell walls [67].

On the other hand, methanolic extracts from the edible primary heads of two globe artichoke varieties, namely “Spinoso Sardo” and “Romanesco Siciliano”, exhibited higher phenolic content values (64.77 and 108.46 g/kg DW, respectively) than those observed in this study for the secondary flower heads of the three genotypes analyzed [58]. As previously discussed, the observed differences could be attributed to several factors, including, among others, the different genotypes, and the various environmental conditions in which they were grown (Sardinia and Sicily for the two cultivars and Central Italy for the three genotypes analyzed in this study). These factors could exert a significant impact on polyphenol biosynthesis and accumulation. According to our findings in the secondary flower heads, 5-O-caffeoylquinic acid (chlorogenic acid) and 3,5- and 1,5-di-O-caffeoylquinic acids were the most abundant phenolic compounds in both cultivars, representing more than 90% of the total polyphenols [58]. In contrast, the flavonoid content, represented only by apigenin and apigenin 7-O-glucoside, was very low (approximately 1% of the total identified polyphenols) [58].

Despite the considerable variability reported in the literature regarding the phenolic content and composition of different artichoke plant parts, the total phenolic compounds identified in the stems and secondary flower heads of two representative genotypes of the “*Carciofo Ortano*” landrace in this study highlight their potential as cost-effective and valuable sources of bioactive compounds, primarily 5-O-caffeoylquinic acid (chlorogenic acid) and 3,5- and 1,5-di-O-caffeoylquinic acids, which, regardless of genotype or plant part, accounted for more than 90% of the total identified polyphenols. These compounds are well-recognized for their potential health benefits, including antioxidant, anti-inflammatory, hepatoprotective, and anticancer properties [76,77,78]. Notably, the highest concentration of these compounds was observed in the stem, a plant part typically discarded as waste. Consequently, stems could serve as an underutilized source of natural antioxidants, with their industrial recovery offering additional economic value for local producers.

In contrast to polyphenols, limited data are currently available on the concentration of the sesquiterpene cynaropicrin in various parts of the artichoke plant. Colantuono et al. [79] reported a cynaropicrin concentration of 2.7 g/kg DW in the stem of the “Tondo di Paestum” variety, which was comparable to the levels of this sesquiterpene lactone found in the same tissue of the two “Carciofo Ortano” genotypes analyzed in this study. Conversely, a significantly lower concentration of cynaropicrin (0.014 g/kg DW) was detected in the primary heads of the same variety compared to the levels found in the secondary heads of the two “Carciofo Ortano” genotypes (0.284–0.977 g/kg DW) in the present study.

Considering that the highest concentration of this sesquiterpene lactone among the various artichoke plant parts has been reported in the leaves, ranging from 4 to 20 g/kg DW [49,79], the two main by-products of artichoke cultivation (stems and leaves) could serve as valuable sources of this important bioactive compound. Several studies have demonstrated that cynaropicrin, which is primarily responsible for the characteristic bitter taste of artichoke, exhibits various biological activities, including anti-hyperlipidemic, anti-inflammatory, and anti-photoaging effects [9,18]. Furthermore, it has been reported that cynaropicrin, even at very low concentrations, can activate the human bitter taste receptor hTAS2R46 in vitro [80]. This property may contribute to the metabolic benefits primarily associated with the consumption of artichoke polyphenols. Indeed, emerging evidence indicates that bitter compounds influence neurohormonal pathways involved in regulating gastrointestinal motility, glucose homeostasis, and appetite control by activating bitter taste receptors distributed throughout the gastrointestinal tract.

#### 3.3.2. Characterization of Volatile Organic Compounds (VOCs) by SPME-GC/MS

The analyses conducted using SPME-GC/MS enabled the identification of numerous volatile components belonging to various chemical classes such as aliphatic alcohols, sesquiterpenes, and monoterpenes (Table 7). These volatiles compounds were found in all analyzed tissues and genotypes, though with varying distributions.

The alcohols 1-butanol, 3-methyl- and 1-butanol, 2-methyl- were present in all investigated samples. The highest percentage of 1-butanol, 3-methyl- was found in the stems of Orte 2 F7 P2 (30.2%) and Orte 1 F4 P10 (24.5%), while lower amounts were detected in the same tissue of Grato 1 (13.2%). On the other hand, 1-butanol, 2-methyl- reached its highest percentage in the SFH of Orte 2 F7 P2 (19%) and in the stem of Orte 1 F4 P10 (16.3%), with lower percentage mean values found in both tissues of Grato 1 (5.0% in the stem and 0.3% in the SFH, respectively). In contrast, 1-hexanol was present in the SFHs but not in the stems of the three investigated genotypes, with the highest percentage mean values detected in Grato 1 (33.8%) and Orte 1 F4 P10 (26.3%). Additionally, significant relative amounts of 3-ethyl-4-methylpentan-1-ol were relieved in the stems of Orte 1 F4 P10 (50.1%) and Orte 2 F7 P2 (48.2%). Finally, 1-pentanol, 3,4-dimethyl- was detected in similar percentages only in the stems of Orte 1 F4 P10 (7.0%) and Orte 2 F7 P2 (6.0%). Among sesquiterpenes, *β*-eudesmene was present in all analyzed samples, but reached significantly higher percentages in the SFHs of all three tested genotypes: 90.8% in Grato 1, 41.1% in Orte 2 F7 P2, and 38.1% in Orte 1 F4 P10 (Table 7). On the other hand, *β*-caryophyllene was detected in both the stem and SFH of Grato 1 (0.7% and 6.7%, respectively), in the SFH of Orte 2 F7 P2 (5.2%) but was completely absent in both tissues of Orte 1 F4 P10. In contrast, the monoterpene *trans-β*-ocimene was present in both the stems and SFHs of Orte 1 F4 P10 (2.1% and 4.7%, respectively) and Grato 1 (24.1% and 0.9%, respectively), but was missing in either tissue of Orte 2 F7 P2 (Table 7).

In general, VOCs have many beneficial effects including antioxidant effects, plant growth stimulation and anti-plant pathogen activity [81]. In particular, sesquiterpene compounds found in these matrices such as, *β*-eudesmene and *β*-caryophyllene are well-known for their antioxidant activity [82,83]. On the other side, branched-chain C5 alcohols such as, 2-methyl-1-butanol and 3-methyl-1-butanol were evaluated for their antifungal activity against *Botrytis cinerea* of tomato and table grape and between the two compounds, 3-methyl-1-butanol was shown as the more active one [84]. In addition, some volatile compounds impart unique flavor and odor characteristics to food. In this regard, 1-hexanol has a fruity/green/sweet aroma [85] while trans-β-ocimene has been reported as one of the compounds that contributed the most to the aroma of organic passion fruit [86].

### 3.4. Cell Viability and Oxidative Stress

The cell viability of differentiated SH-SY5Y cells was assessed using the MTT assay after 24 h of dose-dependent treatments with stem and SFH extracts from the Orte 1 F4 P10, Orte 2 F7 P2, and Grato 1 genotypes. This approach yielded an EC_50_ value of 190.1 µg/mL only for the cells treated with the Orte 1 F4 P10 stem extract. In contrast, no cytotoxicity was observed at the concentrations of treatment considered for the Orte 2 F7 P2 and Grato 1 stem extracts or for the SFH extracts from any of the three genotypes; in these cases, cell viability remained unaffected by exposure to extracts up to 250 µg/mL.

No solvent-related effects were observed at the highest concentration tested.

The neuroprotective potential of the tested extracts was evaluated by exposing pre-treated cells to oxidative stress induced by H_2_O_2_ for 1 h. Pre-treatment was carried out 24 h earlier using five two-fold serially diluted, non-cytotoxic concentrations of stem and SFH extracts, ranging from 0.39 to 6.25 µg/mL for stem extracts and from 12.5 to 200 µg/mL for SFH extracts (Table 8). The stem and SFH extracts from the Orte 1 F4 P10, Orte 2 F7 P2, and Grato 1 genotypes were able to increase the viability of cells stressed by H_2_O_2_. Specifically, in cells exposed to H_2_O_2_, the stem extracts restored cell viability from 29.62% to 49.62% and 34.42% after 24 h of treatment with 6.25 μg/mL of Orte 1 F4 P10 and Orte 2 F7 P2 extracts, respectively. Moreover, the stem extract from Grato 1 increased cell viability from 29.62% to 33.65% after 24 h of treatment with 3.13 μg/mL of extract. Regarding the SFH extracts, those from the Orte 1 F4 P10 and Orte 2 F7 P2 genotypes showed greater activity at a concentration of 100 µg/mL, restoring the cell viability to 50.77% and 45.96%, respectively, while 200 μg/mL of extract was the most active concentration for Grato 1 (46.73% of cell viability) (Table 8).

**Figure 2:**
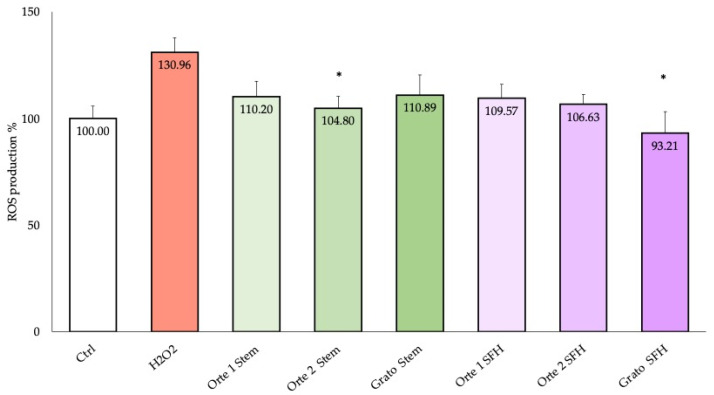
Reduction of H_2_O_2_ induced in intracellular free radical ROS following treatment with stem and SFH extracts of Orte 1 F4 P10, Orte 2 F7 P2 and Grato 1 P3 genotypes. * *p* < 0.001.

The ability to counteract H_2_O_2_ induced intracellular ROS production in differentiated SH-SY5Y cells was investigated by pre-treating the cells for 24 h with two concentrations that showed a significant protection: 6.25 μg/mL for the stem extracts and 200 μg/mL for the SFH extracts. The reduction in free radical ROS following treatment was assessed using the DCFH-DA assay. As reported in Figure 2, the Grato 1 SFH extract led to the highest and most significant reduction in intracellular ROS levels, restoring the values detected in the cells not treated with H_2_O_2_ (Ctrl), followed by Orte 2 F7 P2 stem extract.

As shown in Figure 2, Grato 1 SFH and Orte 2 F7 P2 stem extracts resulted in the highest and most significant reduction in intracellular ROS levels, restoring those of not H_2_O_2_ stressed cells (Ctrl). However, overall, all extracts tested showed activity in reducing intracellular ROS levels (Figure 2).

Since GSH is the most abundant intracellular antioxidant, playing a key role in controlling the production of free radicals formed by oxidative stress, such as that induced by H_2_O_2_, the MCB assay was used to evaluate the effects of stem and SFH extracts on intracellular GSH levels.

**Figure 3:**
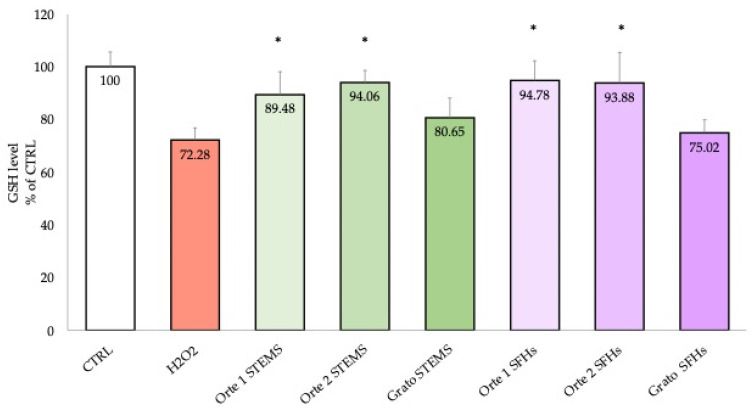
Increased GSH levels following treatment with stem and SFH extracts of Orte 1 F4 P10, Orte 2 F7 P2 and Grato 1 genotypes. * *p* < 0.001.

Differentiated SH-SY5Y cells were pre-treated 24 h earlier with 6.25 μg/mL and 200 μg/mL of stem and SFH extracts, respectively, and then exposed to H_2_O_2_. As shown in Figure 3, the treatment with H_2_O_2_ resulted in a significant reduction in GSH levels compared to control cells. The stem and SFH extracts of Orte 1 F4 P10 and Orte 2 F7 P2 significantly increased GSH levels compared to cells exposed to H_2_O_2_, with values comparable to those of control cells. However, cells treated with Grato 1 stem and SFH extracts did not show a significant increase in GSH levels compared to H_2_O_2_ treated cells.

Oxidative stress plays a key role in the progression of numerous neurodegenerative conditions, characterized by a complex and often reciprocal relationship, where oxidative stress fuels the manifestation of key disease features, which, in turn, exacerbate oxidative stress [87,88].

Neuronal functions are commonly modeled in vitro using cell lines such as differentiated SH-SY5Y cells, which, upon exposure to H_2_O_2_, exhibit metabolic alterations and cell death processes resembling those observed in neurodegenerative diseases [89,90]. The protection against oxidative stress observed following treatment with methanolic extracts from the stems and SFH of the three artichoke genotypes analyzed in this study was correlated with reduced ROS levels and restored GSH levels. To exhibit their antioxidant effects in a cellular model the compounds can break the peroxyl radical chain reactions at the cell membrane surface, or they can be taken up by the cell and react with ROS intracellularly [91].

Among the bioactive compounds present in various parts of the artichoke plant, polyphenols may play a pivotal role in the observed antioxidant activity, particularly 5-O-caffeoylquinic acid (chlorogenic acid) and 3,5- and 1,5-di-O-caffeoylquinic acids, which were the predominant polyphenols identified in the analyzed methanolic extracts. Consistent with our findings, the protective effects of bract and residual leaf extracts against oxidative stress-induced liver damage, evaluated using a hepatocarcinoma cellular model (human HepG2 cells), were primarily attributed to chlorogenic acid, as well as 3,5- and 1,5-di-O-caffeoylquinic acids. Notably, the results demonstrated a strong correlation between polyphenolic content and antioxidant efficacy, with 1,5-di-O-caffeoylquinic acid exhibiting the highest antioxidant activity [92]. Similar findings were reported for a 60% *v*/*v* methanolic extract fraction derived from artichoke by-products (bracts, leaves, and stems), which were particularly rich in hydroxycinnamic acids. This extract significantly reduced ROS production in Caco-2 cells, a model used to evaluate antioxidant capacity against hydrogen peroxide-induced damage in intestinal cells [66].

A restoration of ROS levels after treatment with Grato 1 SFH and Orte 2 F7 P2 stem extracts the values observed in control cells was similarly shown by Jiang and colleagues [93]. They investigated the ability of 4,5-O-dicaffeoyl-1-O-(malic acid methyl ester)-quinic acid (MDCQA) to counteract H_2_O_2_-induced intracellular ROS production in SH-SY5Y cells by DCFHDA assay and observed that ROS generation tended to decrease significantly with pretreatment in a dose-dependent manner, restoring ROS levels to the control value, similarly to the N-acetyl-L-cysteine used as an antioxidant drug [93].

The antioxidant activity of polyphenols present in artichoke can be explained through several mechanisms. Polyphenols are known to prevent lipid peroxidation and scavenge reactive oxygen species (ROS) through their ability to chelate metal ions [94]. Additionally, the antioxidant capacity of caffeoylquinic acids and flavonoids is linked to their hydrogen-donating capacity, which arise from their unique structural features [95]. However, their modulatory effects are primarily associated with the interception of free radicals and ROS within critical signaling pathways involving various transcription factors, protein kinases, and phosphatases [96]. Notably, antioxidants can modulate the nuclear factor E2-related factor 2 (Nrf2) signaling pathway. Studies have shown that polyphenols regulate Nrf2 genetically and epigenetically at transcriptional, post-transcriptional, and translational levels, thereby increasing the cytoplasmic concentration of this protein [97]. The primary role of Nrf2 is to activate the transcription of genes involved in the synthesis of antioxidant enzymes, such as NADPH quinone oxidoreductase, heme oxygenase, catalase, and superoxide dismutase. It also regulates genes encoding enzymes within the glutathione system, including glutathione peroxidase, glutathione S-transferases, γ-glutamylcysteine synthetase, and glutathione synthetase, the latter two being directly involved in glutathione synthesis [98]. Additionally, Nrf2 controls the expression of inflammatory genes such as nuclear factor-kappa B (NF-κB) and transforming growth factor-β (TGF-β) [99]. Interestingly, the cyanopicrin content in artichokes, a compound present in the analyzed tissues of the “Carciofo Ortano” genotypes, may also influence antioxidant activity. This sesquiterpene lactone has been shown to inhibit ROS production by upregulating the transcription of genes encoding Nrf2 and NADPH quinone oxidoreductase [100].

Given that this study has demonstrated the ability of methanolic extracts from artichoke stems and SFHs to mitigate hydrogen peroxide-induced damage in vitro using a cellular model, the therapeutic potential of these extracts may extend beyond the current findings. Further in vivo studies are necessary to explore their broader applications, as illustrated in the following section.

### 3.5. Oral Administration of Artichoke Extracts in D. melanogaster

Recent studies have shown that food supplementation with natural compounds protects the visual system of high-sucrose diet (HSD)-treated flies, improving behavior and reducing neuronal impairment [52,101]. Additionally, a significant reduction in the production of neurotoxic Aβ-peptides was observed in the brains of Alzheimer’s Disease fly models fed with a nutraceutical Lisosan G-enriched diet, which also reduced neuronal apoptosis [102]. Interestingly, these findings revealed that oral administration with natural substances during oxidative stress conditions has beneficial effects on mitochondrial activity, indicating that mitochondrial homeostasis is a suitable parameter for assessing the antioxidant properties of ingested compounds.

**Figure 4:**
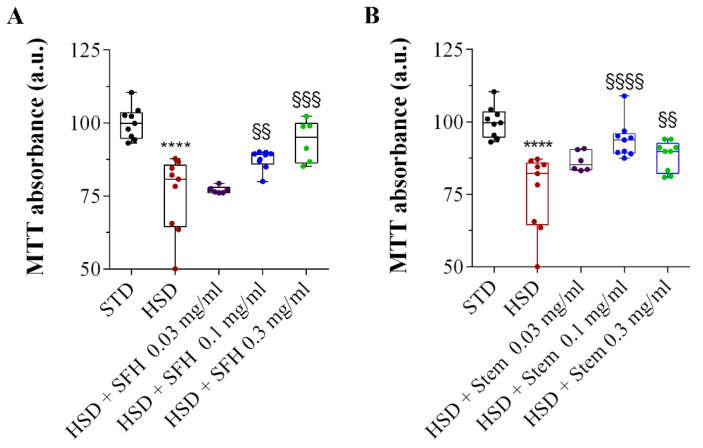
Mitochondrial activity assessment by MTT absorbance in adult flies‘ heads reared on standard diet (STD), high sucrose diet (HSD), or HSD supplemented with 0.03, 0.1, 0.3 mg/mL SFH (**A**) or stem (**B**) extracts. Results are expressed as arbitrary units (a.u.). Data have been obtained from three independent experiments using at least 300 heads for each experimental group. **** *p* < 0.0001 HSD vs. CTRL; §§ *p* < 0.01, §§§ *p* < 0.001, §§§§ *p* < 0.0001 HSD vs. HSD + SFH or HSD + STEM (**B**).

In agreement with previous analyses in head homogenates [52], we found that adult *D. melanogaster* treated with HSD for 10 days displayed impaired mitochondrial activity. Specifically, a decrease in MTT reductive ability was observed in head extract homogenates compared to those from flies on a standard diet (STD) (Figure 4). Remarkably, mitochondrial activity significantly increased when both SFH (Figure 4A) and stem (Figure 4B) extracts were administered in the diet. Interestingly, SFH and stem extracts had a strong positive effect on mitochondrial activity at concentrations of 0.1 and 0.3 mg/mL, but no significant effects were observed at 0.03 mg/mL. These data demonstrate a dose-response effect of artichoke extracts in our in vivo system, improving mitochondrial functionality, with the SFH extract showing a more pronounced trend than the stem extract.

The use of *D. melanogaster* in drug discovery, including the evaluation of bioactive natural compounds, has seen a significant surge in recent decades [102,103,104,105,106], confirming its potential as an in vivo tool that effectively complements traditional vertebrate models. Indeed, the nutrient absorption in *D. melanogaster* occurs similarly to humans [107], making feeding experiments in flies suitable for evaluating the activity and delivery of drugs and other compounds in vivo, including investigating nutraceuticals’ biological effects.

Feeding experiments in *D. melanogaster* were instrumental in supporting the in vitro results on redox homeostasis. Data obtained from HSD flies supported the oral bioavailability and the antioxidant effects of artichoke extracts. Noteworthy, the use of head homogenates suggests a positive neuroprotective role and was particularly significant, as oxidative stress is a common hallmark of hyperglycemic conditions at both systemic and neuronal levels [52,101,108].

## 4. Conclusions

This study highlights the potential of *Cynara cardunculus* L. var. *scolymus* (globe artichoke) landrace, “Carciofo Ortano”, as a valuable source of bioactive compounds with antioxidant properties. A comprehensive analysis of different plant tissues revealed significant variations in antioxidant activity, with stems and secondary flower heads exhibiting the highest values across multiple assays (FRAP, ABTS, DPPH, and FC). HPLC-DAD analysis identified caffeoylquinic acids, particularly 5-O-caffeoylquinic acid (chlorogenic acid), 1,5-di-O-caffeoylquinic and 3,5-di-O-caffeoylquinic acids, as the primary contributors to antioxidant activity. These compounds were further complemented, albeit in low quantities, by the presence of apigenin derivatives and cynaropicrin. Using SPME-GC/MS analysis, VOCs were also detected including alcohols and terpenes with known antioxidant properties. Methanolic extracts from both tissues demonstrated significant antioxidant effects in vitro and in vivo, reducing ROS levels, restoring glutathione balance in neuroblastoma cells, and improving mitochondrial function in *D. melanogaster* under oxidative stress conditions. These findings emphasize their potential neuroprotective and health-promoting properties.

Moreover, the results underscore the untapped potential of artichoke by-products, particularly stems, as sources of bioactive compounds. These insights align with circular economy strategies aimed at minimizing waste and optimizing resource use.

Taken together, the findings highlight the value of artichoke by-products for potential applications in the pharmaceutical and medical sectors, while emphasizing the importance of preserving agricultural biodiversity and fostering innovation for sustainable development. Future studies should focus on developing scalable extraction techniques and conducting clinical validations to fully harness the potential of this artichoke landrace. Enhanced valorization of each plant part and its components could contribute to local socioeconomic development.

## Figures and Tables

**Table 1:** FRAP values of leaf, stem, primary and secondary flower head (PFH and SFH) of six different genotypes of Orte 1 (F4 P10, F3 P8, and F17 P3) and Orte 2 (F7 P2, F7 P5, and F15 P3) populations, along with the four reference genotypes (Campagnano, C3-13, Grato 1 and Castellammare). The ANOVA analysis was conducted within each group (Orte 1, Orte 2, and the four reference genotypes) for all tissues analyzed. Different letters indicate statistically significant differences at *p* ≤ 0.05.

FRAP (µmol TE/g DW)				
Genotype	Leaf	Stem	PFH	SFH
Orte 1 F4 P10	183.57 ± 2.02 ^g^	381.78 ± 1.66 ^b^	270.23 ± 7.91 ^e^	407.52 ± 1.08 ^a^
Orte 1 F3 P8	216.71 ± 1.67 ^f^	400.26 ± 6.02 ^a^	299.02 ± 5.48 ^d^	321.06 ± 0.44 ^c^
Orte 1 F17 P3	114.26 ± 6.13 ^i^	211.29 ± 4.57 ^f^	144.89 ± 2.06 ^h^	254.76 ± 2.09 ^e^
Orte 2 F7 P2	217.54 ± 8.16 ^g^	519.67 ± 5.24 ^b^	357.56 ± 4.21 ^d^	434.14 ± 5.48 ^c^
Orte 2 F7 P5	156.83 ± 2.65 ^h^	311.87 ± 2.90 ^e^	158.21 ± 3.37 ^h^	352.42 ± 1.03 ^d^
Orte 2 F15 P3	243.34 ± 3.09 ^f^	552.57 ± 5.99 ^a^	219.86 ± 0.34 ^fg^	340.80 ± 12.81 ^d^
Campagnano	161.12 ± 1.92 ^i^	329.53 ± 4.64 ^e^	443.31 ± 6.13 ^b^	275.42 ± 1.36 ^g^
C3-13	174.31 ± 8.06 ^i^	403.77 ± 7.49 ^d^	422.94 ± 2.81 ^c^	437.94 ± 1.12 ^bc^
Grato 1	236.29 ± 2.22 ^h^	340.55 ± 0.01 ^e^	429.90 ± 3.37 ^bc^	471.59 ± 0.23 ^a^
Castellammare	173.60 ± 1.40 ^i^	299.52 ± 0.44 ^f^	311.13 ± 6.85 ^f^	334.68 ± 4.25 ^e^

**Table 2:** ABTS values of leaf, stem, primary and secondary flower head (PFH and SFH) of six different genotypes of Orte 1 (F4 P10, F3 P8, and F17 P3) and Orte 2 (F7 P2, F7 P5, and F15 P3) populations, along with the four reference genotypes (Campagnano, C3-13, Grato 1 and Castellammare). The ANOVA analysis was conducted within each group (Orte 1, Orte 2, and the four reference genotypes) for all tissues analyzed. Different letters indicate statistically significant differences at *p* ≤ 0.05.

ABTS (µmol TE/g DW)				
Genotype	Leaf	Stem	PFH	SFH
Orte 1 F4 P10	220.35 ± 1.87 ^f^	380.60 ± 0.75 ^a^	316.54 ± 4.69 ^bcd^	372.16 ± 6.94 ^a^
Orte 1 F3 P8	240.35 ± 4.22 ^ef^	274.38 ± 5.11 ^de^	297.47 ± 2.38 ^cd^	357.55 ± 0.17 ^ab^
Orte 1 F17 P3	147.39 ± 7.38 ^g^	229.63 ± 1.84 ^ef^	339.78 ± 4.07 ^abc^	377.76 ± 0.98 ^a^
Orte 2 F7 P2	260.06 ± 1.85 ^e^	451.64 ± 0.34 ^a^	373.86 ± 6.24 ^bc^	381.98 ± 3.27 ^bc^
Orte 2 F7 P5	214.89 ± 0.61 ^f^	395.45 ± 1.09 ^b^	352.08 ± 0.46 ^cd^	292.56 ± 4.07 ^e^
Orte 2 F15 P3	158.90 ± 5.29 ^g^	383.72 ± 2.75 ^bc^	327.82 ± 1.49 ^d^	369.64 ± 0.06 ^bc^
Campagnano	134.40 ± 5.22 ^ij^	324.98 ± 0.92 ^bc^	391.27 ± 2.18 ^a^	285.46 ± 0.01 ^de^
C3-13	111.53 ± 6.02 ^j^	274.59 ± 2.30 ^e^	258.48 ± 8.40 ^ef^	222.13 ± 1.09 ^fg^
Grato 1	166.78 ± 4.05 ^hi^	388.06 ± 1.78 ^a^	335.08 ± 8.55 ^bc^	381.61 ± 0.57 ^a^
Castellammare	192.13 ± 2.43 ^gh^	279.54 ± 3.10 ^e^	294.30 ± 0.34 ^cde^	360.03 ± 0.11 ^ab^

**Table 3:** DPPH values of leaf, stem, primary and secondary flower head (PFH and SFH) of six different genotypes of Orte 1 (F4 P10, F3 P8, and F17 P3) and Orte 2 (F7 P2, F7 P5, and F15 P3) populations, along with the four reference genotypes (Campagnano, C3-13, Grato 1 and Castellammare). The ANOVA analysis was conducted within each group (Orte 1, Orte 2, and the four reference genotypes) for all tissues analyzed. Different letters indicate statistically significant differences at *p* ≤ 0.05.

DPPH (µmol TE/g DW)				
Genotype	Leaf	Stem	PFH	SFH
Orte 1 F4 P10	27.58 ± 0.07 ^j^	462.18 ± 0.38 ^i^	1075.91 ± 1.76 ^c^	1232.56 ± 4.17 ^a^
Orte 1 F3 P8	978.85 ± 7.67 ^e^	1009.10 ± 4.45 ^d^	1164.42 ± 7.89 ^b^	1017.68 ± 6.59 ^d^
Orte 1 F17 P3	595.15 ± 9.87 ^h^	800.07 ± 7.55 ^g^	825.36 ± 6.28 ^f^	991.80 ± 2.57 ^e^
Orte 2 F7 P2	134.04 ± 6.44 ^k^	1297.61 ± 8.10 ^c^	276.55 ± 5.26 ^j^	1591.16 ± 4.09 ^b^
Orte 2 F7 P5	674.29 ± 1.61 ^h^	1091.79 ± 2.68 ^d^	868.01 ± 4.36 ^f^	1010.87 ± 2.35 ^e^
Orte 2 F15 P3	1102.78 ± 7.69 ^d^	1685.71± 0.03 ^a^	469.36 ± 9.85 ^i^	757.97 ± 0.50 ^g^
Campagnano	379.90 ± 0.13 ^i^	576.63 ± 0.01 ^fg^	562.98 ± 7.55 ^g^	309.98 ± 3.75 ^k^
C3-13	376.94 ± 8.55 ^i^	673.78 ± 0.07 ^e^	584.95 ± 4.79 ^f^	449.16 ± 2.13 ^i^
Grato 1	456.44 ± 0.09 ^i^	1508.29 ± 0.02 ^b^	667.89 ± 2.00 ^e^	1706.41 ± 1.91 ^a^
Castellammare	248.41 ± 6.46 ^l^	476.24 ± 0.06 ^h^	1373.56 ± 9.68 ^c^	928.15 ± 0.05 ^d^

**Table 4:** Total polyphenols content (TPC) expressed as mg GAE/g DW of leaf, stem, primary and secondary flower head (PFH and SFH) of six different genotypes of Orte 1 (F4 P10, F3 P8, and F17 P3) and Orte 2 (F7 P2, F7 P5, and F15 P3) populations, along with the four reference genotypes (Campagnano, C3-13, Grato 1 and Castellammare). The ANOVA analysis was conducted within each group (Orte 1, Orte 2, and the four reference genotypes) for all tissues analyzed. Different letters indicate statistically significant differences at *p* ≤ 0.05.

TPC (mg GAE/g DW)				
Genotype	Leaf	Stem	PFH	SFH
Orte 1 F4 P10	47.16 ± 1.68 ^f^	92.84 ± 1.76 ^b^	46.79 ± 0.04 ^f^	154.27 ± 1.59 ^a^
Orte 1 F3 P8	24.72 ± 1.82 ^g^	58.44 ± 1.2 ^e^	53.99 ± 0.88 ^e^	53.54 ± 1.12 ^e^
Orte 1 F17 P3	47.47 ± 0.76 ^f^	72.91 ± 2.25 ^d^	88.81 ± 1.19 ^b^	79.01 ± 1.25 ^c^
Orte 2 F7 P2	77.01 ± 2.39 ^h^	245.56 ± 1.71 ^a^	119.72 ± 2.25 ^e^	183.44 ± 1.15 ^c^
Orte 2 F7 P5	45.76 ± 1.40 ^j^	221.89 ± 1.08 ^b^	76.74 ± 1.03 ^h^	97.19 ± 1.97 ^g^
Orte 2 F15 P3	65.29 ± 1.72 ^i^	242.17 ± 1.50 ^a^	105.97 ± 3.92 ^f^	173.56 ± 3.38 ^d^
Campagnano	25.36 ± 1.80 ^g^	80.91 ± 1.38 ^c^	126.30 ± 0.99 ^a^	90.82 ± 1.93 ^b^
C3-13	30.82 ± 1.79 ^fg^	34.47 ± 1.61 ^f^	120.36 ± 3.41 ^a^	69.05 ± 0.28 ^d^
Grato 1	47.14 ± 1.64 ^e^	92.09 ± 2.88 ^b^	68.55 ± 4.87 ^d^	97.59 ± 0.77 ^b^
Castellammare	17.11 ± 1.30 ^h^	78.54 ± 2.65 ^c^	49.47 ± 2.58 ^e^	82.77 ± 1.45 ^c^

**Table 5:** Pearson correlation coefficients between the four assays used to evaluate the antioxidant activities of four tissues from ten different artichoke genotypes. Asterisks indicate significance levels: ** *p* < 0.01; * *p* < 0.05.

	FRAP	ABTS	DPPH	TPC
FRAP	1.000			
ABTS	0.659 **	1.000		
DPPH	0.401 *	0.449 **	1.000	
TPC	0.679 **	0.725 **	0.416 **	1.000

**Table 6:** Phenolic compounds identified through HPLC-DAD, expressed as mg/kg DW, for stem and SFH tissues of the three artichoke genotypes analyzed. Nd: no detected. Different letters indicate statistically significant differences among the tissues of the three genotypes at *p* ≤ 0.05 (ANOVA analysis, Tukey test).

	Orte 1 F4 P10		Orte 2 F7 P2		Grato 1	
	Stem	SFH	Stem	SFH	Stem	SFH
1-O-caffeoylquinic acid	145.39 ± 7.18	274.29 ± 3.62	132.66 ± 0.20	226.05 ± 0.68	228.04 ± 0.75	301.49 ± 4.88
3-O-caffeoylquinic acid	360.78 ± 0.73	141.99 ± 3.67	161.16 ± 0.57	151.49 ± 0.47	225.03 ± 0.65	135.35 ± 10.19
5-O-caffeoylquinic acid	9439.31 ± 689.34	7456.31 ± 271.60	6175.45 ± 26.34	7006.45 ± 272.25	6435.69 ± 19.80	7163.26 ± 38.85
Caffeic acid	275.53 ± 9.10	171.56 ± 1.60	134.92 ± 1.46	96.56 ± 0.44	189.15 ± 2.95	150.57 ± 4.03
1,3-di-O-caffeoylquinic acid	31.25 ± 1.40	35.81 ± 0.21	46.70 ± 0.26	35.44 ± 0.02	29.83 ± 0.17	nd
3,5-di-O-caffeoylquinic acid	3922.33 ± 17.20	6655.04 ± 5.63	287,14.31 ± 755.02	19,700.10 ± 30.40	4521.09 ± 238.71	74.26 ± 11.31
1,5-di-O-caffeoylquinic acid	14,397.07 ± 40.98	11,215.88 ± 733.64	52,484.31 ± 74.18	28,600.73 ± 342.99	12,497.05 ± 56.47	6812.23 ± 155.61
Total caffeoylquinic acid	28,571.68 ± 731.92 c	25,950.89 ± 476.78 d	87,849.52 ± 655.32 a	55,816.83 ± 586.41 b	24,125.88 ± 163.82 d	14,637.16 ± 186.66 e
apigenin 7-O-glucoside	nd	166.17 ± 0.35	nd	90.38 ± 1.92	nd	nd
apigenin	nd	nd	nd	44.48 ± 0.71	nd	nd
Total apigenin	nd	166.17 ± 0.35 a	nd	134.86 ± 1.22 b	nd	nd
Total polyphenols	28,571.68 ± 731.92 c	26,117.05 ± 476.426 d	87,849.52 ± 655.32 a	55,951.70 ± 585.19 b	24,125.88 ± 163.82 d	14,637.16 ± 186.66 e
cynaropicrin	283.967 ± 89.72 d	740.99 ± 10.26 c	2254.58 ± 61.95 a	976.85 ± 10.46 b	nd	nd

**Table 7:** Chemical volatile composition (percentage mean value ± SD) of stem and SFH tissues of the three artichoke genotypes analyzed. ^1^ The components are reported according to their elution order on apolar column; ^2^ Linear Retention Indices measured on apolar column; ^3^ Linear Retention indices from literature; nd: not detected.

			Orte 1 F4 P10		Orte 2 F7 P2		Grato 1	
Component ^1^	LRI ^2^	LRI ^3^	Stem	SFH	Stem	SFH	Stem	SFH
1-butanol, 3-methyl-	699	700	24.5 ± 1.15	17.3 ± 0.85	30.2 ± 2.14	17.4 ± 0.23	13.2 ± 0.18	1.2 ± 0.09
1-butanol, 2-methyl-	748	744	16.3 ± 0.25	5.9 ± 0.05	14.2 ± 0.30	19.0 ± 0.86	5.0 ± 0.09	0.3 ± 0.02
1-hexanol	847	841	nd	26.3 ± 1.01	nd	5.9 ± 0.04	nd	33.8 ± 1.02
1-pentanol, 3,4-dimethyl-	1415	1412	7.0 ± 0.08	nd	6.0 ± 0.09	nd	nd	nd
3-ethyl-4-methylpentan-1-ol	1029	1023	50.1 ± 3.20	7.6 ± 0.09	48.2 ± 1.85	11.4 ± 0.10	8.4 ± 0.12	nd
*trans*-*β*-ocimene	1051	1048	2.1 ± 0.02	4.7 ± 0.05	nd	nd	24.1 ± 1.45	0.9 ± 0.08
*β*-caryophyllene	1414	1418	nd	nd	nd	5.2 ± 0.35	0.7 ± 0.06	6.7 ± 0.35
*β*-eudesmene	1488	1481	2.1 ± 0.03	38.1 ± 2.05	1.4 ± 0.09	41.1 ± 2.15	14.8 ± 0.86	90.8 ± 4.50

**Table 8:** Cell viability (%) obtained by MTT assay on differentiated SH-SY5Y cells treated with stem and SFH extracts for 24 h and 1 h with H_2_O_2_. The data are expressed as mean ± SD. Samples vs. Ctrl + (cell viability 29.62% ± 1.04): a, for *p* < 0.0001; b, for *p* < 0.001; ns: not significant.

	μg/mL	Orte 1 F4 P10	Orte 2 F7 P2	Grato 1
Stem	6.25	49.62 ± 1.54 ^a^	34.42 ± 4.07 ^b^	31.54 ± 2.38 ^b^
	3.13	43.65 ± 0.26 ^a^	29.23 ± 3.23 ^ns^	33.65 ± 4.52 ^b^
	1.56	43.46 ± 0.53 ^a^	29.23 ± 2.38 ^ns^	32.31 ± 0.87 ^a^
	0.78	44.04 ± 4.36 ^a^	26.54 ± 3.55 ^ns^	22.69 ± 2.15 ^ns^
	0.39	43.08 ± 3.36 ^a^	25.19 ± 5.99 ^ns^	26.73 ± 0.59 ^ns^
SFH	200	38.08 ± 5.36 ^a^	35.19 ± 3.92 ^b^	46.73 ± 0.01 ^a^
	100	50.77 ± 0.73 ^a^	45.96 ± 6.54 ^a^	43.65 ± 0.26 ^a^
	50	33.65 ± 1.02 ^b^	38.08 ± 1.49 ^a^	35.19 ± 3.04 ^a^
	25	38.08 ± 3.87 ^a^	34.81 ± 5.29 ^b^	29.23 ± 2.21 ^ns^
	12.5	48.27 ± 3.28 ^a^	30.77 ± 1.16 ^ns^	28.08 ± 3.28 ^ns^

## Data Availability

The data contained within the present article and in its Supplementary Materials are freely available upon request to the corresponding author.

## Supplementary Materials

The following supporting information can be downloaded at: https://www.mdpi.com/article/10.3390/antiox14010116/s1, Table S1: List of the ten genotypes used in the analysis; Table S2: Total eigenvalues, relative and cumulative proportion of total variance explained by each component and link of the first two PCs with the antioxidant assays.
