# Supplementary material for: The Role of Extracts of Edible Parts and Production Wastes of Globe Artichoke (Cynara cardunculus L. var. scolymus (L.)) in Counteracting Oxidative Stress
Source: Antioxidants (Basel). 2025 Jan 20;14(1):116. doi: 10.3390/antiox14010116 (PMC11761904; doi:10.3390/antiox14010116)
Supplement: Supplementary file 1 [file antioxidants-14-00116-s001.zip › antioxidants-3413548-supplementary.pdf]

Table S1. List of the ten genotypes used in the analysis.

Genotype	Membership
F3 P8	Orte 1 population
F4 P10	Orte 1 population
F17 P3	Orte 1 population
F7 P2	Orte 2 population
F7 P5	Orte 2 population
F15 P3	Orte 2 population
C3	PGI "Romanesco Artichoke of Lazio"
Campagnano	PGI "Romanesco Artichoke of Lazio"
Castellammare	PGI "Romanesco Artichoke of Lazio"
Grato 1	PGI "Romanesco Artichoke of Lazio"

Table S2. Total eigenvalues, relative and cumulative proportion of total variance explained by each component and link of the first two PCs with the antioxidant assays.

Component	Total eigenvalues	Variance explained	Cumulative variance	Larger correlation with antioxidant assays
1	2.6247	65.617	65.617	ABTS, FRAP, TPC
2	0.6571	16.428	82.045	DPPH
3	0.4092	10.231	92.276	
4	0.3089	7.724	100	
